# The contribution of community health workers to the control of Buruli ulcer in the Ngoantet area, Cameroon

**DOI:** 10.11604/pamj.2013.16.63.1407

**Published:** 2013-10-22

**Authors:** Marius Zambou Vouking, Innocent Takougang, Léonard Mbam Mbam, Lawrence Mbuagbaw, Carine Nouboudem Tadenfok, Claire Violette Tamo

**Affiliations:** 1 Catholic University for Central Africa School of Health Sciences, Yaoundé, Cameroon; 2 Foundation for Health Research and Development, Yaoundé Cameroon; 3 World Health Organization, Yaoundé Cameroon; 4 Center for the Development of Best Practices in Health, Yaoundé Central Hospital, Yaoundé, Cameroon

**Keywords:** Cameroon, community health workers, Buruli ulcer, Mycobacterium ulcerans

## Abstract

**Introduction:**

Buruli ulcer (BU) is a skin disease caused by *Mycobacterium ulcerans*. It is the third most common mycobacterial infection after tuberculosis and leprosy. Community Health Workers (CHWs) hold the potential to support patients and their families at the community level.

**Methods:**

We conducted a cross-sectional descriptive study to assess the participation of CHWs in the early diagnosis and treatment of BU in Ngoantet, Cameroon. The CHWs performance was measured using: the number of cases referred to the Ngoantet Health Centre, the percentage of accomplished referrals, the percentage of cases referred by CHWs confirmed by the staff of Ngoantet Health Centre. Data was analyzed using Epi-info version 3.4.1. and Microsoft Office Excel 2003. The study focused on 51 CHWs in the Ngoantet health area.

**Results:**

The referral rate was 95.0%. Most of the suspicious cases (91.5%) referred were confirmed by health workers. Most CHWs (78.4%) declared that they had identified at least one presumptive case of BU infection.

**Conclusion:**

We conclude that the CHWs can play a key role in scaling up BU control activities using a referral system. This study confirms the role of home visits and inspections in the early detection and treatment of BU.

## Introduction

Neglected Tropical Diseases (NTDs) are the most common infections of the world′s poorest people and a leading cause of chronic disability and poverty in low- and middle-income countries [1]. The NTDs are a group of 17 major disabling conditions that are among the most common chronic infections in the world's poorest people [1, 2]. A guideline for the control or elimination of the seven most prevalent NTDs in Africa (ascariasis, trichuriasis, hookworm infection, schistosomiasis, lymphatic filariasis, trachoma, and onchocerciasis) has been established by a group of private, public, and international organizations working together with pharmaceutical partners and national ministries of health [2, 3].

Buruli ulcer (BU) is a skin NTD caused by *Mycobacterium ulcerans* [1]. It is the third most frequent mycobacterial infection after tuberculosis and leprosy [4]. It is characterized initially by a nodule that later progresses to vast cutaneous ulcerations, mediated by Mycolactone, a toxin secreted by *M. ulcerans* [5]. BU causes significant morbidity in children due to physical incapacity and disfigurement [5]. The exact mode of transmission is the subject of many research activities [1]. Recent evidence suggests that aquatic insects of the genera *Naucoris and Diplonychus* may play a role in disease transmission [1].

BU is endemic in thirty countries, and is suspected to exist in 10 other countries of the African Region [1]. The epidemiologic pattern is defined by the presence of confined foci where BU is endemic [6, 7], with prevalence ranging from a few cases to up to 22% in given communities [8]. The preventive and therapeutic means for reducing the impact of this disease are still very limited [9]. Recently published data suggest that antibiotic therapy with Rifampin and Streptomycin is effective in reducing and even eliminating BU lesions when initiated during the early phases of the disease, decreasing the extent of surgical intervention and drastically reducing recurrence rates [10].

In Cameroon, BU was first described in 1969 in 47 patients in a well confined area located in the neighborhood of the villages of Ayos and Akonolinga, in the valley of the Nyong river [11]. There are currently close to 6000 cases in Cameroon [12]. Only 30% of these patients benefit from medical treatment with antibiotics [12]. Others remain in isolated regions and resort to traditional healing [12]. In 2009, there were 57 new cases of BU in the Ngoantet area, which is a priority zone for control [12]. According to the World Health Organization (WHO), service delivery is the primary function of any health system and entails the provision of “effective, safe, good quality care to those that need it with minimal waste”, [13] and to address health care needs through promotion, prevention, treatment and rehabilitation. Community Health Workers (CHWs) are men or women chosen by the local authorities and trained to provide health services at the community level [14]. CHWs are likely to be more sensitive to their fellow community members’ health problems and to provide to support patients and their families [14].

This study aimed at establishing the contribution of CHWs of the Ngoantet health area in the fight against Buruli ulcer by analyzing their activities and determining their performance.

## Methods

### Study setting

The study was conducted in the Ngoantet area, Centre region, Cameroon where BU is endemic with more than 200 patients reported since 2006 [15]. The Ngoantet health area covers over 419 km^2^ with 13 267 inhabitants distributed in 25 villages. The main economic activity is agriculture and the main commercial product is cocoa. Children aged less than 15 years represent more than 50% of the population. Females represent 55% of the population. The Ngoantet Health area comprises 58 CHWs (33 men and 25 women). The dialogue structures are represented here by the Health and Management Committees whose members are elected by the community. Cases of BU are managed at the Ngoantet Health Center which is the referral center in the Mbalmayo Health District.

### Study design

From April to September 2010, we conducted a cross-sectional descriptive study on the CHWs in Ngoantet area. Fifty-one CHWs were interviewed using a questionnaire with open, semi-open and close-ended questions related to the performance of BU control activities. The study questionnaire was verbally administered to the CHWs by four male interviewers of the same health area. The questionnaires were pre-tested in the near-by Ayos health area on 8 CHWs. We collected socio-demographic data (age and gender); work related data (number of houses visited per week, number of cases detected, number of cases referred); years of working experience, mode of appointment as CHW and other community health activities carried out. We also verified the register at the Ngoantet Health Centre to confirm the reports. The CHWs performance in the control of BU was measured in terms of the number of cases referred to the Ngoantet Health Centre, the percentage of referrals accomplished and the percentage of cases referred by CHWs confirmed by the staff of Ngoantet Health Centre. A confirmed case was defined as a probable case with evidence of *M. Ulcerans*infection, indicated by a Ziehl-Neelsen test for acid-fast bacilli in smears of lesion exudates [16], a positive polymerase chain reaction (PCR) [17] or both. Laboratory analyses were done at the mycobacteriology reference laboratory *Centre Pasteur du Cameroun*, Yaoundé, Cameroon.

**Participants:** All CHWs in the Ngoantet area were eligible to participate, and were included if they consented. We interviewed 51 of the 58 CHWs in the health area.

**Statistical methods:** The data were analyzed using Epi-info version 3.4.1. and Excel 2007. Counts and percentages are reported.

**Ethics:** The study was approved by the ethical committee of the Catholic University and administrative authorization was obtained from the Cameroon Ministry of Public Health. Informed consent was obtained orally and confirmed with thumb prints as the participants were illiterate. All the participants provided informed consent before any interview was conducted.

## Results

### Characteristics of CHWs

The CHWs were aged 25 to 71 with a mean age of 42.1 years. The age range 25-50 was the most represented (92.27%) compared to other age groups. There were more males (64.7%) than females (35.3%) (Table 1). Of 51 CHWs interviewed, 31 (64.7%) reported visiting one to ten homes a month against 16 (31.4%) and 2 (3.9%) who visited (11-20) and (21-30) homes respectively. Each village has about 120 homes. This result shows a good coverage of the different villages by CHWs (Table 1). Of the 51 CHWs investigated; 40 (78.4%) declared to have identified at least one suspected case. CHWs working experience varied between 12 and 48 months. The mean duration of working experience was 36 months. CHWs were either elected by the local community or nominated by the village head or the health area head. The majority were elected (Table 1).

**Table 1:** Other characteristics of community health workers

CHWs characteristics	Frequency N (%)
**Age**	
≤ 25	2 (3.9)
25 – 50	45 (88.3)
> 50	4 (07.8)
**Sex**	
Male	33 (64.7)
Female	18 (35.3)
**Homes visited**	
0	2 (3.9)
1-10	31 (64.7)
11-20	16 (31.4)
21-30	2 (3.9)
**Involvement in other health programs**	
Malaria	33 (68.8)
Immunization	8 (16.7)
HIV/AIDS	7 (14.5)
**Mode of recruitment**	
Nominated by the head of village	3 (5.9)
Elected by community	45 (88.2)
Nominated by the head of the health area	3 (5.9)
**Working experience**	
12 month	2 (4.0)
24 month	7 (13.7)
36 month	33 (64.7)
48 month	9 (17.6)

CHW: community health workers

CHWs activities in the control of BU in Ngoantet

Fifty-nine new suspected cases were identified during the last 12 months. Out of 40 CHWs who declared to have detected suspect cases of BU, 38 (95%) declared to have referred the suspect cases to the Ngoantet Health Center. Of the 40 CHWs who declared to have detected suspect cases of BU, 34 (85%) declared haven referred the forms of first categories to the Ngoantet Health Center (Table 2). The CHWs investigated reported to have identified 59 new cases in the last 12 months out of which 54 were confirmed as cases of BU, with a rate of 91.5% (Table 2). Among the CHWs surveyed, 48 (94.1%) reported having intervened in other health programs such as malaria, HIV/AIDS, Immunization, whereas 3 (5.9%) worked only in the fight against BU (Table 1).

**Table 2:** Distribution of total cases, suspected case and confirmed cases

Cases characteristics	Frequency N (%)
Suspected cases	59 (100)
Confirmed cases	54 (91.5)
**Community health workers characteristics**	
Investigated	51 (100)
Suspected cases identified	40 (78.4)
Referred cases	38 (95.0)
First category cases	34 (85.0)

## Discussion

This study demonstrates the roles of CHWs in the identification and referral of cases of BU. However, it is not without some limitations. Despite checks made at the Ngoantet Health Centre through consultation with monthly activity reports, it was difficult to establish whether all the CHWs provided accurate responses as some responses did not tie with health centre data.

The population structure of Ngoantet is similar to the rest of Cameroon [18] with young with people under fifteen representing 45% of the population. The CHWs were aged 25 to 71 with a mean age of 42. This indicates strong involvement of members of this age in the health area. In addition, there were more male CHWs (64.7%). The women in the Ngoantet health area represent 50.3% of the population live with the organization of traditional society. They are often restricted by household chores and agriculture, and at a disadvantage in the fields of education, health and participation in decision-making. However, the fact that women in Ngoantet are beginning to get involved in the control of BU is a strength for the health area [15]. Other authors have reported that female CHWs may be more reliable [19].

Increased awareness on the efficacy of antibiotics has led to treatment of more patients with both pre-ulcerative conditions and ulcers [20]. The reluctance of some people with BU to seek medical care is consistent with findings in other studies [21, 22]. Traditional therapy was the first choice for treatment for some affected persons because of easy local access, compared to the burden of high transport costs, and loss of income due to absence from work while in medical treatment at a distant site [23]. CHWs identified and referred suspected cases of BU with a 91.5% success rate. Most CHWs (78.4%) had each identified at least one suspected case of BU in the last 6 months. This implies good social mobilization and wide community acceptance that BU is a curable disease that can be managed at the health center. This is indicative of a decline in previous socio cultural perception of the etiology of BU as originating from witchcraft and amenable only to traditional healing.

Our findings show the tremendous impact of health education and community surveillance strategies in BU control. Though this is a laudable community-directed initiative, there is the need for more concerted efforts of the programme to intensify these strategies to reduce BU-related morbidity and increase timely access to medical treatment. This study equally displays the good performance of the referral system in the Ngoantet health area.

CHWs played a major role in the control of BU by referring cases to the health centre

These results confirm the observations of other authors that the CHWs have a crucial role in the fight against BU [13, 15, 21, 24]. Two authors [25, 26] in Ivory Coast found a rate of 67% (cases referred by CHWs confirmed by the staff). This outcome can be explained by a good knowledge of the disease, the large number of home visits and a good referral system in the Ngoantet health area.

As concerns the proportion of pre-ulcerative forms of disease referred by CHWs our results (85%) are higher than those found by Ake et al in Ivory Coast, who found a 38% rate in the Taabo Health District. This could be explained by an intensification of the fight and greater involvement of the Ngoantet community. This early detection could reduce the cost of treatment and length of hospitalization as simple cases are treated at the Ngoantet health center [20–24].

Ninety-four percent of CHWs reported working in other health programs. 33 CHWs (68.8%) are involved in the fight against malaria, 8 (16.7%) are involved in HIV/AIDS activities and 7 (14.6%) were involved in immunization campaigns. The results show that BU is not a priority health problem in the health area despite the fact that 49.01% of the CHWs give 40 to 60% of time of their activities. The fight against BU could be used as leverage to strengthen primary health care in the health area. WHO recommends the need for rehabilitation of patients [27], yet there is paucity of research on its success and integration in the health system.

## Conclusion

CHWs are playing an important role in the fight against BU in the Ngoantet area. Our research was also able to reaffirm the important role of home visits and early detection in the fight against this disease. CHWs should be further empowered and their services used to scale up BU control activities.
